# Reduction of mortality and predictions for acute myocardial infarction, stroke, and heart failure in Brazil until 2030

**DOI:** 10.1038/s41598-020-73070-8

**Published:** 2020-10-20

**Authors:** Camila Alves dos Santos Siqueira, Dyego Leandro Bezerra de Souza

**Affiliations:** 1 Graduate Program in Collective Health, Federal University of Rio Grande do Norte, 1787 Senador Salgado Filho Ave, Lagoa Nova, Natal, RN 59010-000 Brazil; 2 Department of Collective Health, Graduate Program in Public Health, Federal University of Rio Grande do Norte, Natal, Brazil

**Keywords:** Cardiology, Cardiovascular diseases

## Abstract

Cardiovascular diseases (CVD) are responsible for the majority of deaths in Brazil and worldwide, and constitute an important share of non-transmissible diseases. The objective of this study is to analyze the mortality trends of the three main CVD in Brazil and its geographic regions: acute myocardial infarction, stroke, and heart failure. Data predictions until 2030 were also carried out. An ecological study is presented herein, with data for the period 2001–2015. Mortality from these diseases was evaluated by annual trends, and grouped in five-year intervals for the predictions until 2030. All data are publicly available. Acute myocardial infarction was the leading isolated cause of death. Brazilian trends revealed a decrease in the three diseases, with different patterns across geographic regions. The Southeast, South, and Midwest regions presented reductions for the three diseases. The predictions indicated higher rates for men. There was also a reduction in the risk of death from these diseases for Brazil and, despite the different mortality patterns for the three diseases, the Southeast region presents, primarily, lower predicted rates than the other regions. The assessment of trends and predictions for the three main CVD in Brazil revealed general decreasing trends with differences across the geographic regions.

## Introduction

According to the World Health Organization (WHO), non-transmissible diseases are responsible for 71% of deaths in the world^1^ and 74% in Brazil^2^. Non-transmissible diseases include chronic respiratory diseases, cancers, diabetes, mental conditions, and diseases of the circulatory system (also referred to as cardiovascular diseases—CVD)^1^, which are responsible for the majority of disabilities^3^ and deaths worldwide^3,4^. In Brazil, CVD cause 28% of deaths^2^, among which ischemic heart diseases are highlighted as the leading cause, followed by stroke^3,5,6^. Together, these categories account for 1/3 of global deaths^7^.

The risk factors for these diseases include the consumption of tobacco^2,8–12^, arterial hypertension^2,5,6,9,12–15^, inadequate diet^5^, sedentarism, excessive consumption of alcohol, environmental pollution^1,2,5,9^, the presence of metabolic alterations^16^, high cholesterol^3,15,17^, diabetes^6,8,9,11,13^, and overweight and obesity^2,8,9,11,14,15^. Regarding the moderate consumption of alcohol, some findings have suggested lower chances of developing CVD, but no causal conclusions were drawn^13^.

Risk factors can also be classified according to CVD, where dyslipidemia, consumption of tobacco, and diabetes are more associated with ischemic heart diseases. Cerebrovascular diseases are more associated with hypertension^18^ and modifiable factors related to arteriosclerosis^19^.

The assessment of these risk factors is very important^6^, along with analysis of health determinants and social iniquities^8^. Nevertheless, health promotion and prevention actions are also essential, along with the monitoring of trends and their dynamics throughout the years^20,21^. This identifies the regions with higher potential mortality risks due to CVD, and helps plan public policies more effectively. In this context, the objective of this study is to analyze the mortality trends due to the primary CVD (acute myocardial infarction, stroke, and heart failure) in Brazil and its geographic regions, and carry out data predictions until 2030.

## Methods

### Study design

An ecological study is presented herein, with mortality data for the period 2001–2015. The proportion of deaths for each category of Chapter IX of the 10th revision of International Classification of Diseases (ICD) was calculated to determine the primary three CVD. For men and women, the main three CVD were: acute myocardial infarction (I21)—AMI, stroke not specified as haemorrhage or infarction (I64), and heart failure (I50).

After calculations of the main CVD in the period, annual death data, per sex, geographic region, and age groups were obtained from the website of the Department of Statistics of the Brazilian Unified Health System (DATASUS), which originated from the Mortality Information System (SIM). All data are publicly available. Trend assessment employed these data, and for the calculation of predictions, data were grouped in five-year intervals until 2030.

Population data are provided by the Brazilian Institute of Geography and Statistics (IBGE). Census data and inter-census projections were utilized, up to 2012. For the period 2013–2030, population projections were employed, also available from IBGE. Data were selected per age group, for each geographic region, year, and sex. Eighteen age groups were evaluated (five-year intervals), ranging from 0–4 years old to 80–85 years old, with the last age group covering the deaths of people over the age of 85.

In this study, it was calculated the standardized rates for the standard world population. These rates considered a population denominator of 100,000 inhabitants, according to Segi^22^. The rate standardization method is used to correct the effect of different age structures in the analysis performed for different populations. Thus, it enables the comparison of the mortality for the studied diseases in different populations and periods. Even if the populations of each region present different age structures.

Despite improvements in the quality of Brazilian data in recent decades, with broader coverage of SIM and a decrease in the percentage of ill-defined deaths^23–25^, there are differences in data quality across the regions^24^. Data correction^26^ is therefore required for a better estimation^23,24,27^. This study applies a correction to improve the reliability of data, based on a method available at the DATASUS website^28^. According to this method, redistribution of data by sex and age were employed for the correction procedure, along with adjustments regarding the completeness of death records. The difference in percentage terms was calculated in relation to the number of deaths notified to SIM. These data, however, are only available from Chapter IX of ICD-10 and enable the formulation of the correction factor for the ICD factor^28^.

The correction factor calculated for Chapter IX (diseases of the circulatory system) considered each age group, sex, and geographic region, and was applied to data of each selected ICD, respecting the correspondence with age group, geographic region, period, and sex. The factor was calculated for the period 2001–2013, according to the equation: 1 + [(redistributed deaths – notified deaths to SIM)/notified deaths to SIM]. Due to the unavailability of data for the period 2014–2015, the 2013 data correction factor was applied^28^.

The corrected number of deaths was obtained by multiplying the correction factor by the number of deaths for each of the three ICD-10 categories, for the most common CVD, using the equation: Nº of corrected deaths = Nº of deaths per ICD category x correction factor for ICD chapter.

### Trend analysis

Trend analysis used annual data, from 2001 to 2015, and the software *Joinpoint Regression Program*^29^. Analysis of significant variations in the trend curve utilized the annual percentage change (APC), considered for a statistic significance of p < 0.05, which determines the existence of increasing or decreasing rates throughout time. Cases with no statistical significance were treated as “stable”. The number of significant changes in the trend curve indicates the number of joinpoints. This assessment employed the simplest model that justified the changes in slope.

### Predictions

Data prediction considered the addition of annual data for the years constituting each of the five-year intervals (2001–2005; 2006–2010; 2011–2015), with prediction for the subsequent five-year intervals (2016–2020; 2021–2025; 2026–2030), obtaining a mean rate for the quinquennial periods. We use standardized rates per 100,000 inhabitants. The procedure used the statistical package Nordpred, in R software, version 2.8.1, compatible with the aforementioned data packages.

The change occurring between the last observed period (2011–2015) and the last predicted period (2026–2030) was also assessed, according to the changes in death risks for the considered diseases and modifications in the structure and size of population, according to the equation proposed by Möller et al.^30^: Δtot = Δrisk + Δpop = (Nfff—Nooo) = (Nfff—Noff) + (Noff—Nooo).

Where ∆tot is the total variation; ∆risk is the variation due to changes in the risk of death for the evaluated disease; ∆pop is the variation due to changes in population structure and size; Nfff is the number of predicted cases for the last predicted period; Nooo is the number of deaths in the last observed period; Noff is the number of deaths in the last predicted period, maintaining the rates of the last observed period, and Nfff – Nooo is the annual change in the number of deaths.

## Results

Between the years of 2001 and 2015, a total of 2,438,218 deaths were registered in men and 2,209,247 in women due to CVD. This amount represents, respectively, 25.90% and 31.54% of the overall deaths in Brazil for the period, which establishes these diseases as the main causes of deaths in the country.

The three CVD with the higher percentages of deaths were identified, for the same period, as AMI (27.04% in men and 20.75% in women), stroke (13.61% in men and 14.51% in women), and heart failure (8.13% in men and 9.60% in women). AMI is the single disease that caused the most deaths in the country, adding 7% to the total of deaths in the country for men and 6.54% for women.

### Trends

Trend analysis in Brazil is presented in Table 1. For AMI, decreasing trends were identified for men, with APC = -1.7(-2.1;-1.3), and women with APC = -2.2(-2.6;-1.8). Regarding geographic regions, among women, there were reductions for the Southeast, South, and Midwest regions. The North presented stability. For the Northeast, there was a joinpoint in 2006 and another in 2010, with the first period showing increasing rates APC1 = 1.8(0.1;3.5), followed by two stable periods. For men, the North region presented stability. For the Southeast, South, and Midwest regions, reductions were identified. For the Northeast, two joinpoints occurred, in 2006 and 2010, with a stable period, followed by a reduction APC2 = -1.5(-2.5;-0.5) and an increase APC3 = 1.7(1.3;2.2).

**Table 1:** Temporal trend for cardiovascular diseases in Brazil and its geographic regions: Annual Percentage Change (APC), confidence interval, and years with Joinpoints. Brazil, 2001–2015.

	APC 1	95% CI		*p* value	*Joinpoint 1*	APC 2	95% CI		*p* value	*Joinpoint 2*	APC 3	95% CI		*p* value
**Women**														
*Acute myocardial infarction*														
Brazil	− 2.2*	− 2.6	− 1.8	< 0.001	–	–	–	–	–	–	–	–	–	–
North	0.4	− 0.2	1.0	0.2	–	–	–	–	–	–	–	–	–	–
Northeast	1.8*	0.1	3.5	< 0.001	2006	− 2.7	− 6.2	0.9	0.1	2010	0.6	− 1.1	2.3	0.4
Southeast	− 2.7*	− 3.2	− 2.2	< 0.001	–	–	–	–	–	–	–	–	–	–
South	− 4.2*	− 4.8	− 3.7	< 0.001	–	–	–	–	–	–	–	–	–	–
Midwest	− 1.6*	− 2.5	− 0.6	< 0.001	–	–	–	–	–	–	–	–	–	–
*Stroke*														
Brazil	− 5.8*	− 6.3	− 5.3	< 0.001	–	–	–	–	–	–	–	–	–	–
North	− 3.7*	− 4.4	− 3.1	< 0.001	–	–	–	–	–	–	–	–	–	–
Northeast	− 5.8*	− 6.4	− 5.2	< 0.001	–									
Southeast	− 6.6*	− 7.1	− 6.1	< 0.001	–	–	–	–	–	–	–	–	–	–
South	− 5.5*	− 6.2	− 4.9	< 0.001	–	–	–	–	–	–	–	–	–	–
Midwest	− 6.1*	− 7.0	− 5.2	< 0.001	–	–	–	–	–	–	–	–	–	–
*Heart failure*														
Brazil	− 5.5*	− 6.0	− 4.9	< 0.001	–	–	–	–	–	–	–	–	–	–
North	− 5.3*	− 6.1	− 4.5	< 0.001	–	–	–	–	–	–	–	–	–	–
Northeast	− 8.0*	− 9.1	− 6.9	< 0.001	2011	− 1.3	− 6.0	3.7	0.6	–	–	–	–	–
Southeast	− 5.0*	− 5.6	− 4.4	< 0.001	–	–	–	–	–	–	–	–	–	–
South	− 5.5*	− 6.2	− 4.9	< 0.001	–	–	–	–	–	–	–	–	–	–
Midwest	− 6.1*	− 6.9	− 5.4	< 0.001	–	–	–	–	–	–	–	–	–	–
**Men**														
*Acute myocardial infarction*														
Brazil	− 1.7*	− 2.1	− 1.3	< 0.001	–	–	–	–	–	–	–	–	–	–
North	0.9*	0.4	1.3	< 0.001	–	–	–	–	–	–	–	–	–	–
Northeast	1.3*	0.9	1.8	< 0.001	2006	− 1.5*	− 2.5	− 0.5	< 0.001	2010	1.7*	1.3	2.2	< 0.001
Southeast	− 2.4*	− 2.9	− 2.0	< 0.001	–	–	–	–	–	–	–	–	–	–
South	− 3.6*	− 4.2	− 3.0	< 0.001	–	–	–	–	–	–	–	–	–	–
Midwest	− 0.8*	− 1.5	− 0.1	< 0.001	–	–	–	–	–	–	–	–	–	–
*Stroke*														
Brazil	− 5.3*	− 5.7	− 4.9	< 0.001	–	–	–	–	–	–	–	–	–	–
North	− 3.1*	− 3.6	− 2.5	< 0.001	–	–	–	–	–	–	–	–	–	–
Northeast	− 4.8*	− 5.3	− 4.2	< 0.001	–	–	–	–	–	–	–	–	–	–
Southeast	− 6.4*	− 6.9	− 5.9	< 0.001	–	–	–	–	–	–	–	–	–	–
South	− 5.7*	− 6.3	− 5.0	< 0.001	–	–	–	–	–	–	–	–	–	–
Midwest	− 5.9*	− 6.5	− 5.2	< 0.001	–	–	–	–	–	–	–	–	–	–
*Heart failure*														
Brazil	− 5.0*	− 5.6	− 4.5	< 0.001	–	–	–	–	–	–	–	–	–	–
North	− 5.3*	− 6.0	− 4.6	< 0.001	–	–	–	–	–	–	–	–	–	–
Northeast	− 7.3*	− 8.4	− 6.1	< 0.001	2011	0.7	− 4.4	6.0	0.8	–	–	–	–	–
Southeast	− 4.6*	− 5.2	− 4.0	< 0.001	–	–	–	–	–	–	–	–	–	–
South	− 5.5*	− 6.1	− 4.9	< 0.001	–	–	–	–	–	–	–	–	–	–
Midwest	− 6.6*	− 7.3	− 5.8	< 0.001	–	–	–	–	–	–	–	–	–	–

For stroke in Brazil, there was a reduction in men, APC = -5.3(-5.7;-4.9), and women, APC = -5.8(-6.3;-5.3). Regarding geographic regions, there were decreases for all regions, for men and women, with no *joinpoints.*

For heart failure, Brazil presented a reduction for men and women, respectively APC = -5.0(-5.6;-4.5) and APC = -5.5(-6.0;-4.9). Considering geographic regions, reductions were identified for the North, Southeast, and Midwest regions, for both sexes. The Northeast presented a joinpoint for men and women, in 2011, with an initial decreasing period with APC1 = -8.0(-9.1;-6.9) for women and APC1 = -7.3(-8.4;-6.1) for men, followed by stable periods for both sexes.

### Predictions

Tables 2, 3 and 4 presents the corrected number of deaths, divided into age groups (0–19, 20–39, 40–59, and over 60 years old), along with the crude and adjusted rates considering the standard world population for the three primary cardiovascular diseases in Brazil and its geographic regions for the five-year intervals observed (2001–2005; 2006–2010; 2011–2015) and predicted (2016–2020; 2021–2025; 2026–2030).

**Table 2:** Number of deaths due to acute myocardial infarction disease in Brazil and its regions, and crude and adjusted rates per 100,000 inhabitants.

Geographic regions	Men						Women					
	Observed			Predicted			Observed			Predicted		
	2001–2005	2006–2010	2011–2015	2016–2020	2021–2025	2026–2030	2001–2005	2006–2010	2011–2015	2016–2020	2021–2025	2026–2030
**Brazil**												
*Age (years)*												
0–19	404	593	904	949	857	782	123	167	206	295	371	331
20–39	10,734	11,307	12,270	15,355	18,701	22,851	3,737	3,747	3,803	4,391	5,103	6,269
40–59	71,728	77,040	79,590	83,974	87,830	95,719	31,228	33,528	34,132	36,000	37,669	41,088
≥ 60 years	151,608	169,050	193,707	219,752	251,891	287,489	124,149	137,032	157,529	177,542	201,111	231,274
Total	234,474	257,990	286,471	320,030	359,279	406,841	159,237	174,474	195,670	218,228	244,255	278,962
Crude rate	53.69	55.41	58.51	62.04	67.57	74.78	35.35	36.17	38.75	41.19	44.57	49.59
Standardized rate	68.47	61.07	57.57	54.24	51.43	49.67	37.01	31.93	29.44	27.47	25.74	24.75
**North**												
*Age (years)*												
0–19	55	100	85	97	96	92	16	20	30	26	25	24
20–39	796	1,027	1,176	1,404	1,598	1,811	242	287	326	432	490	502
40–59	3,331	4,339	4,834	5,744	6,400	7,241	1,360	1,594	1,826	2,157	2,539	3,049
≥ 60 years	6,507	8,524	11,258	13,752	17,150	20,683	4,179	5,339	7,209	9,208	11,801	14,704
Total	10,689	13,990	17,353	20,998	25,244	29,827	5,797	7,240	9,391	11,822	14,855	18,279
Crude rate	30.45	36.06	40.73	45.70	52.18	59.15	16.95	19.09	22.61	26.38	31.29	36.72
Standardized rate	53.35	55.08	57.85	59.00	58.34	56.62	28.93	28.15	29.72	30.65	30.51	29.83
**Northeast**												
*Age (years)*												
0–19	218	310	373	394	395	376	57	96	88	88	83	76
20–39	3,369	4,085	4,483	5,241	5,573	5,976	1,192	1,304	1,272	1,247	1,158	1,024
40–59	15,664	18,552	20,276	22,970	25,251	27,395	8,640	9,930	10,100	10,641	10,979	11,675
≥ 60 years	39,286	46,864	55,462	61,806	71,967	83,402	32,920	40,156	47,789	53,892	61,937	71,645
Total	58,537	69,811	80,594	90,411	103,185	117,149	42,809	51,486	59,249	65,866	74,156	84,420
Crude rate	48.25	54.03	59.64	64.23	71.86	80.49	33.95	38.32	42.08	44.80	49.05	54.68
Standardized rate	61.75	62.7	63.51	63.59	62.26	60.58	36.68	36.43	35	33.44	31.5	30
**Southeast**												
*Age (years)*												
0–19	95	146	373	256	238	222	28	48	80	62	57	53
20–39	4,662	4,455	4,828	5,792	6,983	8,299	1,605	1,511	1,596	1,849	2,166	2,425
40–59	36,553	38,001	37,923	38,324	38,779	42,178	14,550	15,407	15,630	16,439	17,294	19,339
≥ 60 years	70,958	76,972	86,524	98,189	111,624	126,807	59,428	63,369	71,954	80,727	91,212	105,947
Total	112,268	119,574	129,648	142,561	157,625	177,505	75,611	80,335	89,260	99,076	110,729	127,765
Crude rate	60.67	61.04	63.32	66.19	71.13	78.44	39.16	39.04	41.88	44.60	48.45	54.74
Standardized rate	72.89	62.27	56.98	52.16	48.73	47.05	37.19	30.79	28.13	26.25	24.91	24.54
**South**												
*Age (years)*												
0–19	25	30	29	28	26	24	9	3	4	3	3	3
20–39	1,164	984	992	1,091	1,175	1,258	447	415	382	409	402	337
40–59	11,284	11,008	10,884	10,770	10,764	11,630	4,824	4,660	4,360	4,243	4,343	4,925
≥ 60 years	26,001	26,952	28,286	30,978	34,349	38,898	21,695	22,019	22,765	23,990	25,881	29,291
Total	38,474	38,974	40,191	42,867	46,313	51,810	26,975	27,097	27,511	28,645	30,629	34,555
Crude rate	59.70	57.52	57.26	58.14	61.08	66.93	40.84	38.83	38.13	37.96	39.38	43.40
Standardized rate	72.61	58.16	50.23	44.04	40.15	38.63	39.61	30.54	25.44	21.67	19.37	18.52
**Midwest**												
*Age (years)*												
0–19	19	20	24	23	22	21	13	6	8	7	7	7
20–39	646	786	852	1,065	1,190	1,342	243	244	235	250	219	218
40–59	4,475	5,109	5,777	6,592	7,403	8,427	1,792	1,981	2,286	2,612	2,920	3,192
≥ 60 years	7,743	9,636	12,448	15,421	19,011	22,940	5,114	6,255	8,243	10,728	13,727	17,444
Total	12,883	15,551	19,101	23,101	27,625	32,730	7,162	8,486	10,772	13,597	16,873	20,860
Crude rate	41.78	45.79	51.65	57.77	65.25	73.73	23.08	24.62	28.85	33.63	39.21	46.01
Standardized Rate	61.97	57.14	57.09	55.98	54.72	53.66	33.63	28.78	28.52	28.02	27.28	26.73

**Table 3:** Number of deaths due to stroke in Brazil and its regions, and crude and adjusted rates per 100,000 inhabitants.

Geographic regions	Men						Women					
	Observed			Predicted			Observed			Predicted		
	2001–2005	2006–2010	2011–2015	2016–2020	2021–2025	2026–2030	2001–2005	2006–2010	2011–2015	2016–2020	2021–2025	2026–2030
**Brazil**												
*Age (years)*												
0–19	214	287	213	186	199	138	180	193	130	112	87	56
20–39	2,884	2,631	2,014	1,961	2,007	2,543	2,296	1,781	1,348	1,081	995	1,138
40–59	24,389	20,655	16,540	13,245	11,720	11,874	18,087	15,111	11,688	8,601	7,248	7,125
≥ 60 years	111,215	109,749	101,002	93,718	91,513	94,506	110,284	111,087	101,849	94,223	90,836	94,004
Total	138,702	133,322	119,769	109,110	105,438	109,061	130,847	128,172	115,015	104,018	99,166	102,324
Crude rate	31.76	28.63	24.46	21.15	19.83	20.04	29.05	26.57	22.78	19.63	18.1	18.19
Standardized rate	40.15	30.63	23.36	17.76	14.06	11.95	28.82	21.73	15.85	11.72	9.09	7.65
**North**												
*Age (years)*												
0–19	63	55	39	49	48	46	24	38	28	34	33	31
20–39	368	405	326	355	355	430	273	224	189	145	121	121
40–59	2,145	2,020	1,780	1,578	1,507	1,544	1,626	1,467	1,154	852	703	696
≥ 60 years	7,864	8,899	9,259	9,113	9,555	10,355	6,516	7,607	7,656	7,590	7,900	8,568
Total	10,440	11,379	11,404	11,095	11,465	12,375	8,439	9,336	9,027	8,620	8,756	9,416
Crude rate	29.75	29.33	26.77	24.15	23.7	24.54	24.68	24.61	21.74	19.24	18.45	18.91
Standardized rate	53.48	45.58	39.1	32.45	27.13	23.55	41.27	35.21	27.69	21.77	17.26	14.44
**Northeast**												
*Age (years)*												
0–19	102	144	122	94	70	57	95	92	60	48	40	36
20–39	1,308	1,089	852	802	787	876	1,023	740	544	388	294	276
40–59	7,836	6,524	5,356	4,552	4,160	4,213	7,140	5,582	4,082	2,825	2,300	2,220
≥ 60 years	43,958	40,675	36,755	32,114	31,165	32,166	43,174	41,365	37,003	32,297	30,151	30,305
Total	53,204	48,432	43,085	37,562	36,182	37,311	51,432	47,779	41,689	35,558	32,785	32,837
Crude rate	43.86	37.49	31.88	26.68	25.2	25.63	40.79	35.56	29.61	24.18	21.69	21.27
Standardized Rate	52.33	40.33	32.13	25.49	20.77	17.99	40.71	30.86	22.26	16.15	12.23	10.12
**Southeast**												
*Age (years)*												
0–19	49	66	47	54	51	48	38	49	31	38	35	33
20–39	1,010	843	603	541	490	492	792	579	455	366	336	318
40–59	10,859	8,758	6,540	4,862	4,139	4,288	6,997	5,794	4,400	3,273	2,816	2,973
≥ 60 years	42,645	40,524	36,157	32,827	31,425	32,386	44,449	42,558	37,870	34,288	32,868	34,530
Total	54,563	50,191	43,347	38,284	36,105	37,214	52,276	48,980	42,756	37,965	36,056	37,853
Crude rate	29.49	25.62	21.17	17.77	16.29	16.44	27.07	23.8	20.06	17.09	15.78	16.22
Standardized rate	35.8	25.73	18.58	13.36	10.26	8.69	24.48	17.36	12.37	9.01	7.06	6.13
**South**												
*Age (years)*												
0–19	25	30	29	28	26	24	6	13	8	10	9	8
20–39	1,164	984	992	1,091	1,175	1,258	169	170	114	105	74	57
40–59	11,284	11,008	10,884	10,770	10,764	11,630	2,041	1,705	1,495	1,203	1,145	1,180
≥ 60 years	26,001	26,952	28,286	30,978	34,349	38,898	16,473	16,826	15,818	15,604	15,897	17,672
Total	38,474	38,974	40,191	42,867	46,313	51,810	18,689	18,714	17,435	16,921	17,124	18,917
Crude rate	59.7	57.52	57.26	58.14	61.08	66.93	28.29	26.81	24.16	22.42	22.01	23.76
Standardized rate	72.61	58.16	50.23	44.04	40.15	38.63	26	19.44	14.56	11.28	9.25	8.32
**Midwest**												
*Age (years)*												
0–19	11	11	10	11	10	10	21	6	5	6	5	5
20–39	169	187	155	155	149	118	149	105	69	66	57	53
40–59	1,422	1,224	1,051	909	933	1,123	1,028	851	687	557	507	497
≥ 60 years	5,345	5,357	5,169	4,906	4,925	5,289	4,395	4,559	4,619	4,748	5,108	5,886
Total	6,947	6,779	6,385	5,981	6,018	6,539	5,593	5,521	5,380	5,377	5,677	6,441
Crude rate	22.53	19.96	17.26	14.96	14.21	14.73	18.03	16.02	14.41	13.3	13.19	14.21
Standardized rate	34.8	25.14	19.11	14.35	11.54	10.18	25.87	18.14	13.74	10.5	8.44	7.36

**Table 4:** Number of deaths due to heart failure in Brazil and its regions, and crude and adjusted rates per 100,000 inhabitants.

Geographic regions	Men						Women					
	Observed			Predicted			Observed			Predicted		
	2001–2005	2006–2010	2011–2015	2016–2020	2021–2025	2026–2030	2001–2005	2006–2010	2011–2015	2016–2020	2021–2025	2026–2030
**Brazil**												
*Age (years)*												
0–19	1,138	813	572	524	458	368	865	542	353	302	246	214
20–39	3,615	3,197	2,499	2,167	1,950	1,999	1,885	1,435	1,120	907	801	774
40–59	13,399	12,152	10,487	9,378	8,868	9,176	8,971	7,934	6,865	6,132	5,784	6,150
≥ 60 years	65,996	62,567	59,752	57,372	59,195	65,164	76,490	71,772	70,219	69,244	72,122	80,702
Total	84,148	78,729	73,310	69,440	70,470	76,706	88,211	81,683	78,557	76,585	78,952	87,841
Crude rate	19.27	16.91	14.97	13.46	13.25	14.1	19.58	16.93	15.56	14.45	14.41	15.61
Standardized rate	23.86	17.79	14.14	11.27	9.46	8.53	18.92	13.55	10.63	8.6	7.3	6.67
**North**												
*Age (years)*												
0–19	234	168	96	96	81	68	185	92	68	57	49	44
20–39	465	470	334	280	235	224	208	153	112	109	98	91
40–59	992	935	770	681	650	696	500	426	424	444	485	537
≥ 60 years	4,354	4,005	3,927	3,541	3,590	3,908	3,342	2,994	3,110	3,162	3,530	4,166
Total	6,045	5,578	5,127	4,597	4,557	4,896	4,235	3,665	3,714	3,771	4,162	4,837
Crude rate	17.22	14.38	12.03	10	9.42	9.71	12.38	9.66	8.94	8.42	8.77	9.72
Standardized rate	29.64	21.41	17.16	13.18	10.68	9.37	19.7	13.58	11.33	9.53	8.35	7.75
**Northeast**												
*Age (years)*												
0–19	636	443	295	282	247	197	470	279	160	137	108	91
20–39	1,536	1,312	1,050	959	857	875	885	621	440	373	328	327
40–59	4,194	3,276	2,790	2,645	2,709	2,986	3,235	2,380	1,870	1,561	1,399	1,476
≥ 60 years	22,711	18,264	16,703	14,189	13,815	14,475	22,446	17,988	16,971	15,218	14,837	15,447
Total	29,077	23,295	20,838	18,075	17,629	18,531	27,036	21,268	19,441	17,288	16,672	17,341
Crude rate	23.97	18.03	15.42	12.84	12.28	12.73	21.44	15.83	13.81	11.76	11.03	11.23
Standardized rate	27.86	19.04	15.35	12.19	10.19	9.16	20.97	13.54	10.36	7.98	6.44	5.63
**Southeast**												
Age (years)												
0–19	233	167	130	122	105	83	203	124	95	82	66	57
20–39	1,226	1,066	825	726	710	759	615	469	407	343	309	299
40–59	5,768	5,468	4,876	4,249	3,831	3,792	3,648	3,467	3,121	2,870	2,784	2,991
≥ 60 years	25,795	26,298	25,767	26,133	27,682	31,059	33,375	32,627	32,751	33,374	35,391	40,360
Total	33,022	32,999	31,598	31,230	32,327	35,694	37,841	36,687	36,374	36,669	38,551	43,708
Crude rate	17.85	16.85	15.43	14.5	14.59	15.77	19.6	17.83	17.07	16.51	16.87	18.73
Standardized rate	21.39	16.62	13.34	10.82	9.22	8.37	17.26	12.7	10.21	8.54	7.47	7.01
**South**												
Age (years)												
0–19	54	36	31	27	30	25	30	34	22	23	19	16
20–39	206	161	152	174	177	213	130	118	93	77	90	111
40–59	1,602	1,459	1,259	1,189	1,167	1,256	1,097	1,030	943	873	820	861
≥ 60 years	10,330	9,823	9,553	9,780	10,393	11,659	13,773	13,710	13,104	13,181	13,827	15,652
Total	12,192	11,479	10,995	11,170	11,767	13,153	15,030	14,892	14,162	14,155	14,755	16,640
Crude rate	18.92	16.94	15.67	15.15	15.52	16.99	22.75	21.34	19.63	18.76	18.97	20.9
Standardized rate	23.42	16.73	13.25	10.73	9.2	8.48	20.42	15.04	11.53	9.24	7.84	7.22
**Midwest**												
*Age (years)*												
0–19	58	46	39	32	26	21	33	29	16	22	21	21
20–39	378	312	213	148	132	157	138	105	79	62	47	40
40–59	1,246	1,125	854	651	538	497	707	659	512	420	368	383
≥ 60 years	4,298	4,335	3,808	3,342	3,163	3,250	3,926	4,118	3,990	4,000	4,284	4,948
Total	5,980	5,818	4,914	4,173	3,859	3,925	4,804	4,911	4,597	4,503	4,719	5,391
Crude rate	19.4	17.13	13.29	10.44	9.11	8.84	15.48	14.25	12.31	11.14	10.97	11.89
Standardized rate	29.11	21.23	14.56	10	7.42	6.11	22.2	15.91	11.71	8.79	7.03	6.19

The predictions indicate a reduction in Brazilian rates, for the three studied diseases, for men and women. The geographic regions, however, present different patterns. For AMI, the South and Southeast presented a more pronounced reduction and lowest rates for the predicted periods, for men and women. For stroke, the lowest rates occurred for the Southeast and Midwest, for the observed and predicted periods, for men and women. For heart failure, among men, the lowest predicted rates occurred in the Southeast and Midwest, and for women, the lowest rates occurred in the Midwest and Northeast.

The ratio between the rates of men and women is always higher than one, except for the prediction of the period 2026–2030 for the Midwest region, where the female rate is higher. Among the cardiovascular diseases studied, AMI presents the most significant difference between men and women, surpassing the 2:1 ratio in different regions and periods.

**Figure 1:**
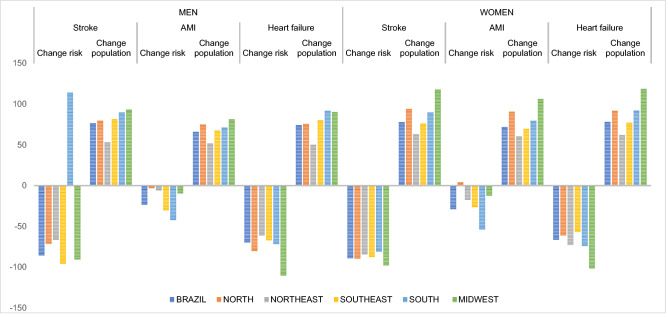
Change in death risk and the in structure and size of population, between the last observed period (2011–2015) and the last predicted period (2026–2030).

Another assessment carried out from the predictions is the change in the risk of death by the studied diseases in comparison with changes in population structure and size. Comparison of periods 2011–2015 and 2026–2030 revealed, for Brazilian data, a reduction in the absolute number of deaths due to stroke, and increases for AMI and heart failure for men and women. For the three diseases, there was a reduction in the risk of falling ill from each evaluated disease. The increase verified for AMI and heart failure was justified by changes in population structure and size (Fig. 1).

For the geographic regions, Fig. 1 depicts an increase in the death risk due to stroke in men of the South region. Also, there was a slight increase in the death risk due to AMI in women of the North region. For the remaining regions, there were reductions in the risk of falling ill due to all the CVD studied, with changes in the number of deaths justified by changes in population structure and size.

## Discussion

The study of mortality trends for the main CVD enabled the identification of reductions in the Brazilian rates for the three evaluated diseases. This reduction was also evidenced by other CVD studies that employed different methods^31–35^ and corroborated by studies that applied joinpoint assessment^36,37^.

The predictions indicated a decreasing risk of death due to these diseases in Brazil, for the predicted data. Concerning geographic regions, varied patterns were verified among the diseases, with better perspectives of reduction for the regions with higher socioeconomic levels^31,35,38^. This could be related to factors such as social development, risk behavior, access and coverage of the health system in each region^35,39,40^, as Brazil has continental dimensions and evident regional inequalities^39,41,42^. Another important result was the higher mortality in men, which has also been reported by other studies^24,32,35^.

Despite the decrease in rates, an increase is expected in the number of deaths due to AMI and heart failure. This finding is explained by the aging process of the Brazilian population, with older age groups being more affected by these diseases^4,5,8,21^. In this way, although decreasing rates were identified, this process implies in the necessity of a better structure of health systems to address the situation^34^, especially in more critical regions, with worse access to healthcare^34^. Therefore, population aging has generated several demands towards the health system, considering the necessities of reducing the burden of the disease and promoting healthy aging^32,33,36,39,43^.

Regarding stroke, a reduction was observed in the rates and in the number of deaths. This could be due to the evolution of the fight against modifiable risk factors for this disease and improvements in treatment. Scientific literature indicates a similar result with developed countries, where better conditions exist for prevention and treatment^3,43^. However, similar results were also found amongst African countries^12^.

Another important finding is that mortality due to ischemic heart diseases surpassed that of cerebrovascular diseases in Brazil in recent decades. Other studies report similar results in Brazil for 1980–2012^18^ and 1990–2016^19^, associated with broader access to diagnosis and treatment of systemic arterial hypertension, the primary risk factor for cerebrovascular diseases^18^. Nevertheless, cerebrovascular diseases cause a significant impact on populations. It is estimated that, in 2015, approximately 9 million first-time brain accidents occurred in the world^7^ with 5.5 million deaths in 2016, while 116.4 million people recovered with side-effects in the same year^19^.

This study also identified AMI as the single disease that caused the most deaths in Brazil, in men and women. This disease is also the primary cause of death in the world and the main cause of health loss (except for Sub-Saharan Africa). In 2015, the estimative was 7.3 million heart attacks, with 100.6 million people living with arterial heart diseases^7^.

Despite being the single leading cause of deaths in Brazil, decreasing mortality trends were identified for AMI during the studied period, except for the Northeast region for men, where there were 2 joinpoints, with a increasing period after 2010. The Southeast, South, and Midwest regions presented reductions in rates for men and women. This finding is probably associated with a better structure of the health system in these regions and better access to healthcare^32^. Data from 2012 show that important health coverage indicators are better in these three regions, with a higher number of medical appointments per inhabitant, higher proportion of the population covered by health plans, and higher proportion of people that had a medical consultation in the previous 12 months. Resource indicators must also be mentioned, such as a higher number of hospital beds per inhabitant, and higher average expense per hospitalization^44^.

Heart failure must also be highlighted as the disease with the highest number of deaths among those under 20 years of age, possibly associated with congenital heart issues. The adverse effects of heart failure include morbidity and costs of treatment and hospitalizations, as well as difficult recovery^19,45^.

When analyzing regional differences in CVD mortality in Brazil, the three primary cardiovascular diseases present reductions for the Midwest region. However, it is expected that female rates surpass male rates for the Midwest region regarding heart failure, in the predicted period 2026–2030. Decreasing trends were revealed for the rates of the South and Southeast regions, for the three main CVD studied herein, which has already been reported by scientific literature^31,32,35,38^. These results can be explained by better conditions for the diagnosis and treatment in these geographic regions, which concentrate the highest gross domestic products and human development indices of the country and imply in better survival conditions^35,46^.

Besides, the Southeast and Suth regions present a better healthcare structure for the attention to chronic diseases. These regions present the highest life expectancies within Brazil, with healthier lifestyles, besides being the wealthiest. These factors reflect differences in the access and quality of health services across geographic regions^38,47^. The North and Northeast regions face additional difficulties such as poverty, lack of good quality education, and unplanned urbanization, which can negatively impact cardiovascular health, leading to unfair distribution of income, resources, and power^3,35^. These conditions can be associated with the increasing period identified for AMI in the Northeast, among men, after 2010.

The North and Northeast regions present the highest predicted rates for the three diseases studied. The different Brazilian regions experience different stages of the epidemiological transition, and more impoverished areas face more challenges to control and prevent non-transmissible chronic diseases^35,47^.

In this context, the WHO established a global action plan (2013–2020) with priorities directed to the prevention and control of non-transmissible chronic diseases. The priorities outlined include the reduction of risk factors, promotion of health, and mapping of areas with the occurrence of these diseases. Social, economic, behavioral, and politic determinants must be known to guide the public policies and measures aimed at preventing and controling these diseases^20^.

Nevertheless, more stringent objectives were stipulated during a 2015 meeting that involved several countries, with the creation of the “Sustainable Development Goals – SCG”, based on the development objectives of the millennium. One of the goals established is directed to health and wellbeing, which is to reduce 1/3 of non-transmissible diseases through prevention and treatment, while also promoting mental health and wellness^48^. These global goals were established to reduce the impact of these diseases, but it is also fundamental to control and reduce the exposure to risk factors throughout the years. These diseases are better confronted when lifestyle habits are improved, with reductions in the consumption of sugar and alcohol^47^. A higher exposure to risk factors can cause an increase in future rates^8,9,42^.

Other authors have remarked on the importance of a healthy diet, emphasizing its impact on the health and prevention of CVD^15,34,49^. Some of the unhealthy habits that cause CVD include the high consumption of sodium, trans fats, cholesterol, and salt, along with the low consumption of fiber, fruit, vegetables, nuts, seeds^3,12,15,17,49^, and omega 3@@@^49^. In contrast, the presence of flavonoids in the diet presents an inversely proportional association with cardiovascular disease mortality^50^.

In Brazil, reduction of the exposure to CVD risk factors has occurred through the control of hypertension, diabetes, dyslipidemias, obesity^2,20,35,38,42^ and tobacco prevention^32,34,38,42^. The country has promoted better access to the medical treatment of these conditions and has been stimulating the practice of more healthy lifestyles, which include physical activity and campaigns against tobacco, through public policies^2,20,35^. The broader access to the medical treatment of hypertension, diabetes, and dyslipidemias started in 2004 with the creation of the “popular pharmacy” program, aimed at the universalization of access to medicines^51^. The program was extended and experienced changes related to financing and destination of funds^52^. Despite the good results obtained, a better access to treatment is not sufficient, on its own, to guarantee adhesion. Activities directed to the promotion of health and education of the population are also necessary.

Regarding the control and prevention of tobacco use, Brazil has promoted legislative and educative measures. In this context, the actions of the National Agency of Sanitary Vigilance (ANVISA) are crucial to promote better health, regulations, control, and inspection of products related to tobacco. In 1999, within ANVISA, the administration of tobacco-derived products was created, and, throughout the following years, different regulations were elaborated to restrict the advertising and use of such products^53^.

Besides the measures related to control the consumption of tobacco, the Federal Government launched the Health Academy Program in 2011 to promote the practice of physical activity, healthy eating habits, and positive changes in lifestyle. Within the program, primary attention plays a vital role in health promotion and prevention activities^54^ and stimulates healthy habits in the population. However, there are still no studies that confirm the results of this program concerning CVD.

The strengthening of public policies for the prevention, treatment, and vigilance of CVD must carefully consider regional differences. Studies focusing on the trends and predictions of mortality rates enables actions to be planned and redirected. These studies support planning efforts and the creation of new health policies, along with the assessment and improvement of existing policies guided to the promotion and prevention of health^33,43,47,55^ and best allocation of health-destined funds^47^.

A limitation of this study, which is inherent to ecological studies, is the impossibility of establishing causal inferences due to the use of aggregate data. In this context, the level of regional data coverage and completeness of SIM could have influenced the results of trends. Also, data from the ICD-10 chapter (diseases of the circulatory system) was employed for the calculation of the correction factor, as there are no data corrected by category for ICD-10 in DATASUS. However, this limitation is minimized because this study considers the three diseases with the highest mortalities and takes into account separation by sex and age group, which provides a more trustworthy correction factor.

In conclusion, the assessment of trends and predictions for the three main CVD in Brazil revealed general decreasing trends. This national decrease, however, includes differences across the geographic regions. Regions with higher purchase power present better perspectives for the reduction of mortality in future studies. In this sense, governmental policies must be strengthened, especially regarding the control of modifiable risk factors, focusing on reaching the goals stipulated by WHO and SDG.
